# The Effects of Military Occupation on Semen Analysis

**DOI:** 10.7759/cureus.101546

**Published:** 2026-01-14

**Authors:** Rebecca W Gregg, James K Aden, Tanya L Glenn

**Affiliations:** 1 Department of Obstetrics and Gynecology, Brooke Army Medical Center, San Antonio, USA; 2 Graduate Medical Education, San Antonio Uniformed Services Health Education Consortium, San Antonio, USA

**Keywords:** environmental exposure, infertility, male factor, military occupation, semen analysis

## Abstract

Introduction

Infertility is becoming increasingly common, with a large contribution from male factor infertility. The majority of active-duty service members are men in their prime reproductive years. There are factors unique to the military that could impact fertility, including occupational, traumatic, and psychological exposures. However, there is limited research in the military population that analyzes a potential link between occupational exposures and the risk of male infertility.

Material and methods

A retrospective review of semen analyses was conducted between 2020 and 2022 at a large military treatment facility in the United States. The occupational code for each individual was queried from the Defense Manpower Data Center Reporting System (DMDCRS). Patients were excluded if their semen analysis was conducted after receiving a vasectomy or chemotherapy. If multiple semen analyses for a single individual were collected, the most favorable was selected.

Results

A total of 1008 semen analyses were reviewed, of which 551 semen analyses met the inclusion criteria. There was no significant difference in sperm concentration, total motile count, or the number of individuals with low spermatozoa concentration (≤15 million/mL) between the occupational groups (p=0.77). A secondary analysis found that trainer pilots had significantly lower spermatozoa concentrations compared to fighter pilots (p= 0.028).

Conclusions

This study did not find an increased risk of male infertility in any of the occupational groups. Strengths of this study include the large number of semen analyses included, focusing on the major portions of the semen analysis associated most with the risk for male infertility, and utilizing the DMDCRS to associate with potential work exposures, reducing risk for reporting bias. Weaknesses of the study include its retrospective nature, which could introduce selection bias given a mixture of individuals undergoing semen analysis for infertility or other reasons, the fact that semen analysis is a surrogate marker only for infertility, and that this was only conducted at a single military base. Different bases/missions may lead to variable exposures that could be associated with infertility. Future studies should increase the number of military bases included and consider longitudinal outcomes such as pregnancy or live birth rates. Assessing and minimizing the impact of military occupational exposures on the risk for male infertility is of particular importance due to the significant financial and emotional burden associated with the evaluation and treatment of infertility.

## Introduction

Infertility is defined as failure to establish a clinical pregnancy after 12 months of regular, unprotected intercourse in females under the age of 35 years [1]. This condition affects an estimated 8-20% of couples in the United States, and male factors are thought to contribute to a third of infertility issues [2,3]. Multiple genetic and lifestyle factors are known to contribute to infertility, but approximately 30% of cases are still considered unexplained [4].

Currently, there are 1.3 million active-duty service members across all branches of the military, of which 92% are under the age of 40, thus performing their service during peak reproductive years [5]. Spousal separation because of time spent on deployments and high-demand missions can pose challenges to fertility.

There are additional factors unique to the military population that could impact fertility, including occupational, traumatic, and psychological exposures. The impact of combat environments on male fertility is not well understood, although studies have shown that psychological stress has a negative impact on semen parameters, including volume, concentration, motility, and morphology [6]. Additional military occupational exposures include physical exposures, such as heat and radiation, and chemical exposures, such as pesticides and solvents [6]. In an otherwise young, healthy, active-duty male, military occupational exposures may threaten current and future reproductive potential. Prior studies of males in the military found higher rates of male infertility among pilots and air crew, thought to be a result of radiation and physical job demands [7-9].

The objective of this study was to determine the association between military occupation and the risk for abnormal semen analysis.

This article was previously presented as a meeting abstract at the 2023 Armed Forces District Meeting in September 2023.

## Materials and methods

This was a retrospective review of semen analyses and military occupation collected between 2020 and 2022 at Brooke Army Medical Center, a large military treatment facility located in San Antonio, Texas, United States. The study was approved as Non-Research Determination by the Brooke Army Medical Command Human Research Protections Office.

Samples and data collection

A total of 1008 semen analyses were reviewed. Data values collected included semen pH, motility, concentration, and volume. An additional categorical variable for normal/low sperm counts was created, with low sperm count set at <15 million/mL as defined by the World Health Organization (WHO) [10]. The Department of Defense Identification Number (DOD ID) associated with each semen analysis was also collected. Patients were excluded if their semen analysis was conducted after receiving a vasectomy or chemotherapy.

**Figure 1:**
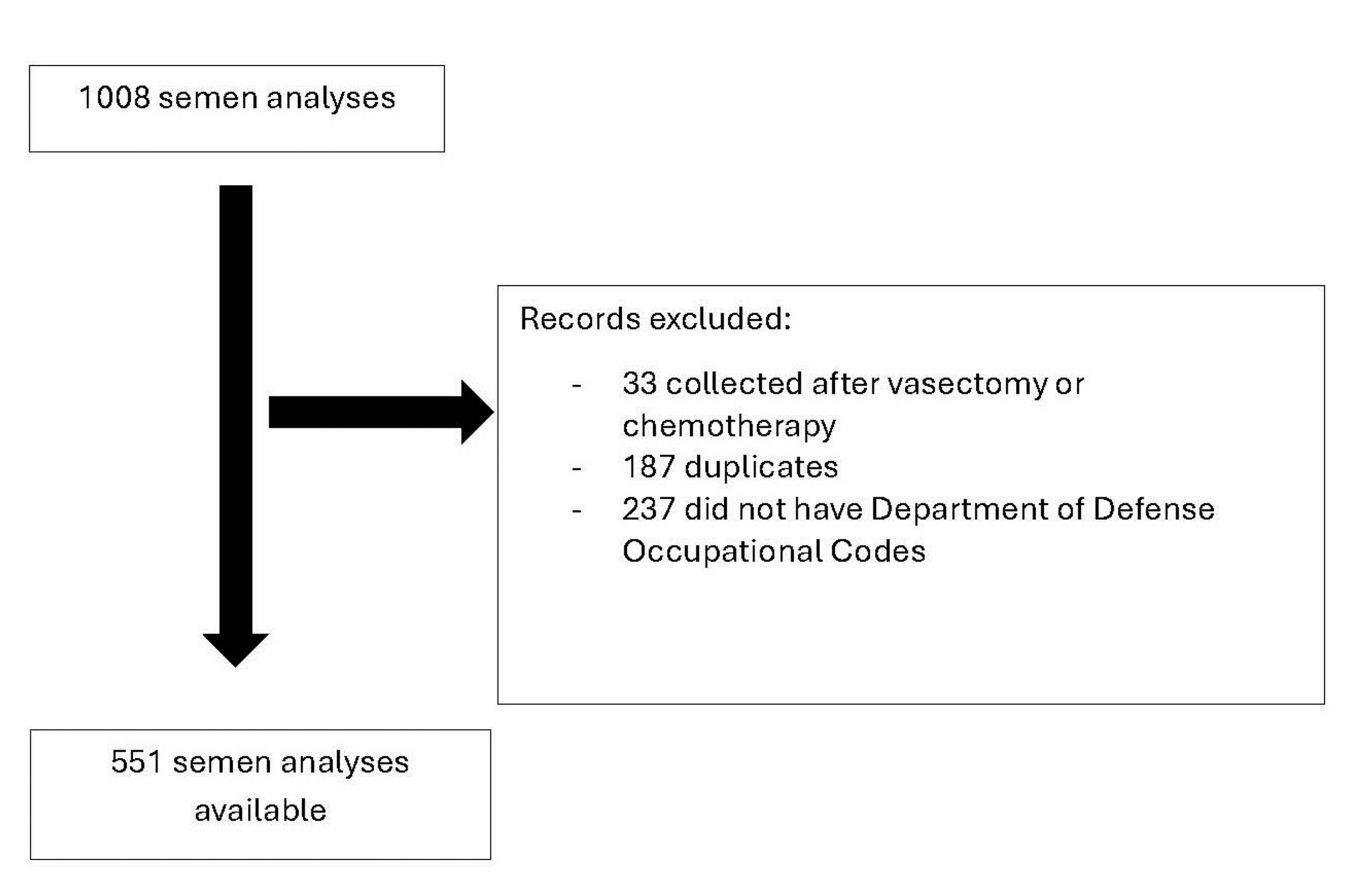
Flow diagram for inclusion and exclusion criteria

Using the DOD ID, the Department of Defense Occupational Code for each individual was queried from the Defense Manpower Data Center Reporting System (DMDCRS). Of the individuals who had completed semen analyses, 237 did not have a Department of Defense Occupational Code in DMDCRS and were also excluded. For patients with multiple semen analyses, the analysis with the highest total motile count was included, as a single abnormal semen analysis could reflect a recent illness and not a chronic sperm issue. No semen analysis data were missing. A total of 551 semen analyses ultimately met the inclusion criteria (Figure 1).

The Department of Defense Occupational Code was used to identify the military department in which the individual worked. These departments were then organized into more general occupational groups for analysis. For pilots, the Service Occupational Code was used to identify their status as either a trainer pilot or a fighter pilot, as previous studies noted higher rates of infertility in pilots. 

Data analysis

The 551 semen analyses that met the inclusion criteria had sperm data extracted, including semen pH, motility, concentration, and volume, which were summarized using means and standard deviations (SDs). Analysis of semen analysis results for each occupational group was compared using the Kruskal-Wallis test. A secondary analysis comparing trainer pilots to fighter pilots was also conducted using the Kruskal-Wallis test. The number of individuals in each occupational group with low sperm counts was compared using Pearson’s chi-squared test. Box and whisker plots were created, showing the median, first, and third quartiles within the box. The lines or whiskers extended 1.5 times the interquartile range (IQR) from the top and bottom of the box. If the data did not extend to the end of the whiskers, then the whiskers extended to the minimum and maximum data values. All statistical analyses were considered statistically significant if p<0.05. All analysis was performed using JMP v 14.0 (SAS Institute Inc., Cary, North Carolina, United States).

## Results

**Figure 2:**
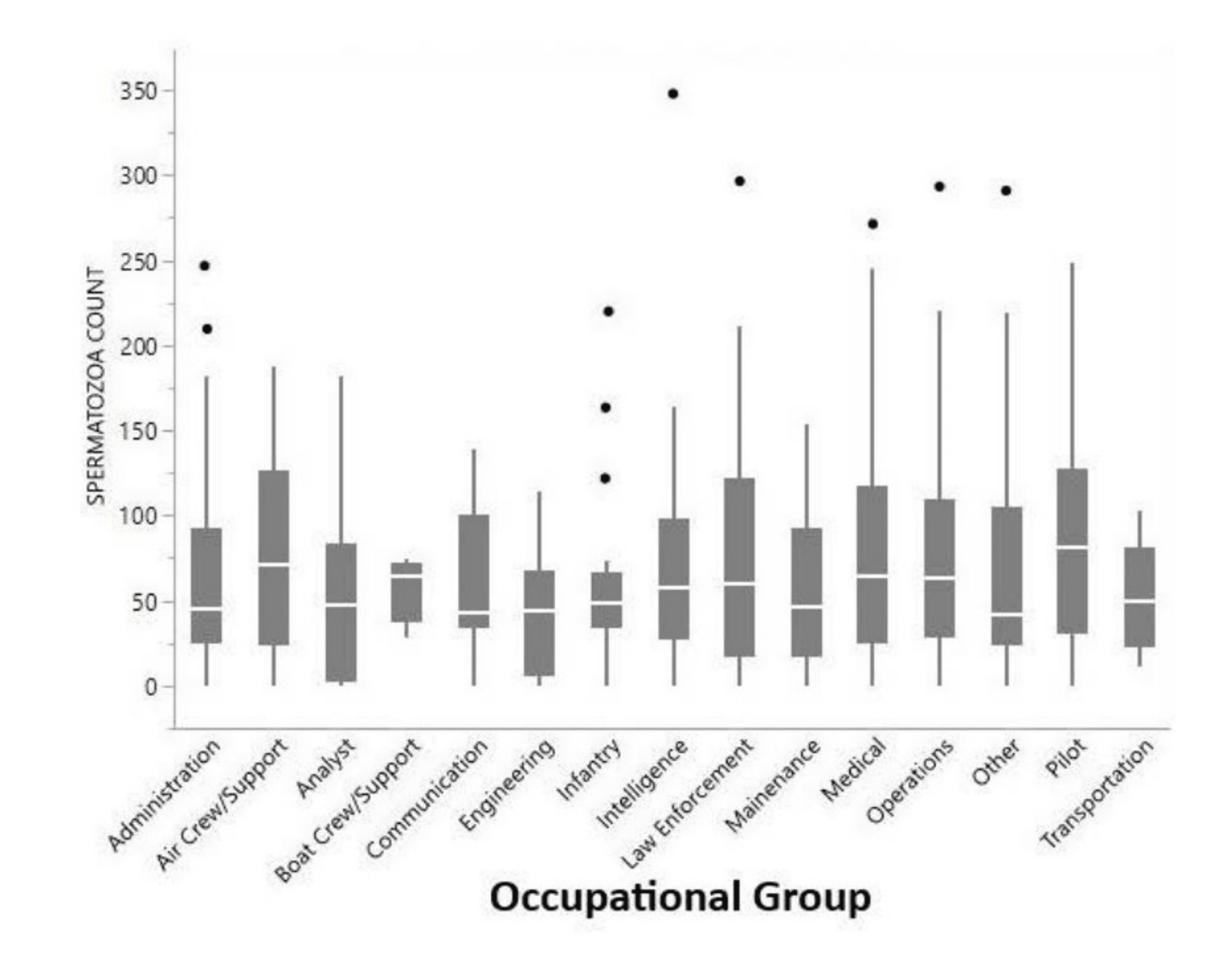
Distribution of spermatozoa concentration by occupational groups No significant difference was noted in mean spermatozoa count (Kruskal-Wallis test χ2 = 9.90, p=0.769) or the number of individuals with spermatozoa count ≤15 million/mL (Chi-Squared test χ2 = 10.75, p=0.705).

A total of 551 semen analyses were included in this study and divided into 15 broad occupational groups. Univariate analysis of sperm concentration by occupational group did not find any significant differences in distributions between the groups (p=0.77). The number of individuals with low spermatozoa concentration (≤15 million/mL) was not significantly different across the occupational groups (p=0.70) (Figure 2). Similarly, no significant difference in total motile count was seen across occupational groups (p=0.11). Total motile count was calculated by multiplying concentration by volume by motility.

**Figure 3:**
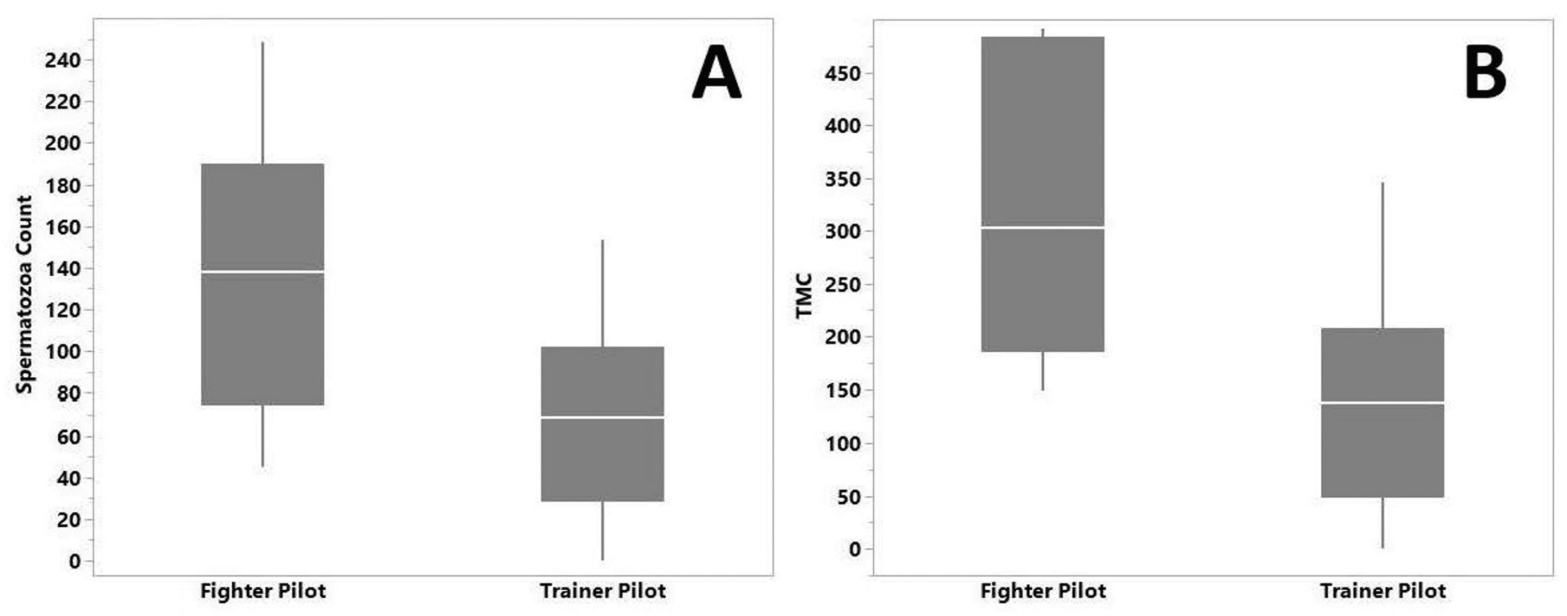
Distribution of spermatozoa concentration and total motile count (TMC) by pilot type. (A) Box plot analyzing the mean spermatozoa concentration in trainer pilots versus fighter pilots; trainer pilots have a lower spermatozoa concentration (Kruskal-Wallis test χ2 = 4.84, p=0.028). (B) Box plot comparing total motile count (TMC) for trainer pilots versus fighter pilots, where trainer pilots have a lower mean TMC (Kruskal-Wallis test χ2 = 6.09, p=0.014).

A secondary analysis comparing fighter pilots with trainer pilots found a statistically significant difference in spermatozoa concentration. Trainer pilots had significantly lower spermatozoa concentrations compared to fighter pilots (p= 0.028) (Figure 3A). The mean spermatozoa concentration was 66.7 ± 46.0 million/mL and 137.4 ± 70.9 million/mL for trainer and fighter pilots, respectively. Similarly, trainer pilots had significantly lower total motile counts than fighter pilots (Mean 139.8 ± 102.4 million versus 320.3 ± 152.2 million, p=0.014) (Figure 3B). There was no significant difference between trainer pilots and fighter pilots in the number of individuals with low spermatozoa concentration (≤15 million/mL).

## Discussion

The goal of this study was to assess the association between military occupations and male infertility risk. The results found that none of the categorized military occupations were associated with a higher risk of male infertility. Specifically, a comparison of spermatozoa concentration or total motile count by occupational group did not show a statistically significant difference.

The secondary analysis found that fighter pilots were over two-fold higher in spermatozoa count and nearly threefold higher in total motile concentration versus training pilots. One hypothesis for this difference is the frequency and number of hours spent flying as a training pilot. Depending on the type of aircraft a pilot is trained on, they could fly 16 hours per month or more [11], which decreases to 5.7-8.1 hours per month for fighter pilots [12]. The potential decrease in radiation exposure may account for the higher spermatozoa concentrations in this population since sperm lifespan is 70-80 days [13]. In contrast, Kuebker et al. found that Air Force pilots were disproportionately affected with infertility (p<0.0001) by comparing observed rates of infertility to expected rates based on the total number of service members in each group [7]. They also noted that observed rates of infertility were significantly higher than expected for both trainer pilots (p=0.001) and fighter pilots (p=0.03). While our study did not find that pilots as a group had a significant difference in spermatozoa concentration and total motile count compared to other occupational groups, trainer pilots demonstrated lower spermatozoa concentrations and total motile count than fighter pilots. Although this difference was statistically significant, the clinical significance is likely negligible as neither of these groups meets the WHO standard for a low sperm count of ≤15 million/mL, which has been associated with male infertility.

This study did not find an increased risk of male infertility in any of the occupational groups. This includes groups that would be hypothesized to have higher risk occupational exposures, including pilots, air crew, and infantry, due to radiation exposure, physical demands, and other occupational hazards unique to these positions. Our results are not consistent with prior studies. A 2011 retrospective study by Kuebker et al. compared rates of infertility among 7519 male Air Force personnel between 2001 and 2009 using Air Force Specialty Codes (AFSC) [7]. The diagnosis of infertility was based on International Classification of Diseases, Ninth Revision (ICD-9) diagnostic codes for male factor infertility. They found that individuals who worked in Aerospace Maintenance, Helicopter Maintenance, and Integrated Avionics systems were disproportionately affected with increased rates of infertility. Similarly, the observed rates of infertility were higher for trainer pilots (p=0.001), mobility pilots (p=0.02), and fighter pilots (p=0.03). Multiple other studies have attempted to assess the effects of specific occupational exposures, such as radiofrequency electromagnetic fields, psychological trauma, and genitourinary injuries [14-18]. However, many of these studies rely on questionnaires, surveys, and diagnosis coding and should be interpreted with caution due to human error and recall bias. 

To our knowledge, this is the first study assessing male infertility risk due to military occupation by analyzing semen analysis. The study by Kuebker et al, which evaluated the impact of military occupational exposures on male infertility, used ICD-9 diagnostic codes [7]. On the other hand, the current study evaluated the distribution of spermatozoa concentration and total motile count amongst each occupational group, allowing for more accurate criteria for defining the risk for male factor infertility by current guidelines. One of the strengths of this study was the utilization of specific components of a semen analysis to define what is considered abnormal from the WHO guidelines, as opposed to relying on coding or questionnaire data. However, this can only be used as a surrogate for the risk of male factor infertility. Additionally, the use of Department of Defense Occupational Codes enabled accurate reporting of occupational data without risk of reporting bias. Yet, as there were 153 unique Department of Defense Occupational Codes included in this study, they had to be placed into 15 broader categories to allow for meaningful comparisons. There may be significant differences in possible occupation exposures within each group that were unable to be appreciated, given the current study design. All aircraft types were grouped together in one category, but it is known that some aircraft have increased radiation exposure [19]. The occupational codes also only reflect the current status of the military member, not necessarily their previous exposure if they were in a different occupation or had exposures from prior deployments.

There has been a paucity of research into male infertility due to the perception that assisted reproductive technology (ART) has “solved” the issue of male infertility. This topic is of particular importance due to the high costs associated with fertility treatments. TRICARE benefits include basic infertility evaluations and treatments leading up to ART. Although not a TRICARE-covered benefit, there are significantly reduced costs of ART for service members and their families, as the costs of physician fees, ultrasounds, and laboratory work at specific military bases are covered [20]. 

Although all semen analyses collected between 2020 and 2022 at Brooke Army Medical Center were included in the study, excluding duplicates and those collected after chemotherapy or vasectomy, there is a risk of selection bias. The rationale for collecting a semen analysis could not be obtained; however, this laboratory study is commonly ordered from the urology or gynecology departments in the setting of an infertility evaluation. Given that this population would be at higher risk of infertility, the semen analysis results may be skewed towards lower concentrations and motility counts. Future studies evaluating the impact of military occupational exposures on the risk for male infertility should be conducted in an unselected male population to reduce the risk of selection bias. Additionally, when an individual had multiple semen analyses collected, the best result was the one included in this study, which would skew the results towards the null. However, this was determined to be the optimal value, as including the worst result may reflect recent illness and not a chronic issue with the individual’s sperm. Last, information concerning the patient’s body mass index, nicotine use, chronic illness, duration of potential exposures, or exposures outside of the military setting could not be obtained, all of which are known to have a negative influence on semen analysis results.

The impact of the coronavirus disease 2019 (COVID-19) pandemic also cannot be undermined. Although the pandemic greatly affected the hours that civilian pilots were able to fly, it had less of an impact on military pilots. At the same time, military pilots have seen a decline in the number of flight hours per month over the last decade [12,21]. The impact of COVID-19 on semen analysis has been studied extensively, which saw alterations in sperm concentration, motility, and/or morphology depending on the study [22,23]. Additionally, some studies showed sperm parameters improved after recovery, while others did not [23,24]. This could have skewed our results in a negative fashion, potentially increasing the number of abnormal semen analysis results overall; however, given our negative results, it is highly unlikely that this impacted our conclusion.

## Conclusions

Although male factors are thought to contribute to a third of infertility issues, research into male infertility has been limited. This study did not find an association between any specific military occupational groups and decreased spermatozoa concentration or total motile sperm. Assessing and minimizing the impact of military occupational exposures on the risk for abnormal semen analysis and possible male factor infertility is of particular importance due to the significant financial and emotional burden associated with the evaluation and treatment of infertility. This study was limited by its retrospective design at a single institution, and the utilization of semen analysis as surrogate for the risk of male factor infertility. Future studies should consider the utilization of a randomly selected patient population to determine if military occupational exposures do have an impact on semen analysis, as well as longitudinal studies to demonstrate if abnormal semen analysis results in male factor infertility.
